# Determining Occurrence Dynamics when False Positives Occur: Estimating the Range Dynamics of Wolves from Public Survey Data

**DOI:** 10.1371/journal.pone.0065808

**Published:** 2013-06-19

**Authors:** David A. W. Miller, James D. Nichols, Justin A. Gude, Lindsey N. Rich, Kevin M. Podruzny, James E. Hines, Michael S. Mitchell

**Affiliations:** 1 United States Geological Survey, Patuxent Wildlife Research Center, Laurel, Maryland, United States of America; 2 Pennsylvania State University, Department of Ecosystem Science and Management, University Park, Pennsylvania, United States of America; 3 Montana Fish, Wildlife and Parks, Helena, Montana, United States of America; 4 United States Geological Survey, Montana Cooperative Wildlife Research Unit, University of Montana, Missoula, Montana, United States of America; University of Alberta, Canada

## Abstract

Large-scale presence-absence monitoring programs have great promise for many conservation applications. Their value can be limited by potential incorrect inferences owing to observational errors, especially when data are collected by the public. To combat this, previous analytical methods have focused on addressing non-detection from public survey data. Misclassification errors have received less attention but are also likely to be a common component of public surveys, as well as many other data types. We derive estimators for dynamic occupancy parameters (extinction and colonization), focusing on the case where certainty can be assumed for a subset of detections. We demonstrate how to simultaneously account for non-detection (false negatives) and misclassification (false positives) when estimating occurrence parameters for gray wolves in northern Montana from 2007–2010. Our primary data source for the analysis was observations by deer and elk hunters, reported as part of the state’s annual hunter survey. This data was supplemented with data from known locations of radio-collared wolves. We found that occupancy was relatively stable during the years of the study and wolves were largely restricted to the highest quality habitats in the study area. Transitions in the occupancy status of sites were rare, as occupied sites almost always remained occupied and unoccupied sites remained unoccupied. Failing to account for false positives led to over estimation of both the area inhabited by wolves and the frequency of turnover. The ability to properly account for both false negatives and false positives is an important step to improve inferences for conservation from large-scale public surveys. The approach we propose will improve our understanding of the status of wolf populations and is relevant to many other data types where false positives are a component of observations.

## Introduction

Presence-absence surveys have become increasingly prominent in large-scale ecological and conservation research [1], [2]. Occurrence data have the advantage of being relatively easy to collect and can be related to many important ecological processes such as habitat use, range-dynamics, metapopulation dynamics, and occupancy-abundance relationships [1]. Particularly important from the standpoint of understanding ecological processes is the ability to use empirical data to estimate occupancy transition probabilities (i.e., colonization and extinction) and to investigate how occupancy dynamics are affected by dynamics of habitat and co-occurring species [1], [3]. New methods for estimating species occurrence probabilities have opened the door to utilizing large-scale occurrence data collections, many of which have engaged the public in the data collection process. Utilizing the public can expand the scope and scale of data collection by orders of magnitude as compared to typical research efforts [4], [5]. However, observation error is likely to be an especially significant issue for these types of data, meaning they should be approached with proper caution [6]–[10] If inferences are to be reliable it is necessary to account for observation uncertainty, including both non-detection and misidentification [11].

Ecologists have long recognized the need to account for imperfect detection when estimating parameters for wildlife populations and have developed an extensive set of methods to deal with non-detection [12]. Recent effort has focused on the need to also account for misclassification and misidentification when estimating population parameters. For example, adaptations of traditional mark-recapture models have focused on various types of classification uncertainty [13]. Additionally, the availability of analytical techniques to deal with individual misclassification have increased the utility of techniques that identify individuals using genetic identifiers [14] and visual patterns [15].

Similarly, most attention for studies of species occurrence have focused on non-detection [1], although recent efforts have also considered misclassification errors. In occupancy studies, misclassification happens when sites that are unoccupied are recorded as being occupied. These false positive errors are common in many occurrence sampling methods [7], [11], [16]–[18]. This is problematic because, when unaddressed, even small rates of false positive errors can result in substantial bias in single season estimators of occupancy [19], [20] and estimators for colonization and extinction rates [11], [21]. Detection errors are often ignored when public surveys are analyzed and, when addressed, effort has generally focused on false negative errors [6], [10]. Previous attempts to address misclassification have largely focused on ad hoc methods to try to reduce their occurrence in data sets [7], [22].

Two approaches have been suggested for estimating occupancy when false positives occur in single season occupancy analyses. The first is a simple modification of the standard occupancy estimator [23], which allows for false positive detections to occur at unoccupied sites [19]. Observed numbers of detections at sites are treated as a binomial mixture of the true positive detection probability at occupied sites and false positive detection probability at unoccupied sites. Miller et al. [20] extend this to deal with cases where detections can be divided into uncertain detections, which have some probability of being a false positive, and certain detections, which are assumed to have zero probability of being a false positive. Their approach reduces bias and increases accuracy when this added information is available about the certainty of observations. Hanks, Hooten & Baker [24] demonstrated a similar formulation for using Bayesian hierarchical methods. Both Royle & Link [19] and Miller et al. [20] also discuss parameterizations for situations where multiple occupancy states occur.

We extend previous methods which allow for false positive errors in single season data to models used to estimate occupancy dynamics across multiple seasons. To illustrate the approach, we examine occupancy dynamics for gray wolves (*Canis lupis*) from 2007–2010 in Montana. We estimate site level extinction and colonization probabilities to determine how relative occupancy differs across habitat types, whether wolves are still expanding their range in this area, and how frequently the occupancy status of sites changes. We compare the results from our estimates that account for false positives to estimates from two methods that do not. The first is where detection errors are assumed not to occur (no false negatives or false positives) and the second is where only non-detections errors are assumed to occur (false negatives occur but no false positives).

## Methods

### Statistical Model

Miller et al. [20] described a general approach to obtaining single season estimates of occupancy when false positive detections occur. We extend this approach to estimate occupancy dynamics across multiple seasons where the probability a site is in a given occupancy state is governed by a Markov process. We focus on the special case where detections can be divided into those that are certain (i.e., probability that a detection is a false positive is zero) and uncertain detections.

There are two possible occurrence sampling designs where both certain and uncertain detections could be recorded. The first is where either certain or uncertain detections can occur during a single sampling occasion. For example during an avian point count survey, observers may consider visual observations of morphologically cryptic species uncertain because of the potential for misidentification, but auditory observations certain if the call is distinct. The second sample design occurs when only one observation type may occur during any given sampling occasion so that detections during a sampling occasion are either all uncertain or are all certain. As an example, consider a mammal species where both scat-surveys and direct-trapping occurs. For many species, the probability of false positives occurring for scat surveys will be non-trivial due to potential species misidentification, and thus we would want to deem detections by this method uncertain. Our sampling design could be used if a second survey type that could be considered certain, such as trapping and direct handling, occurred in at least a subset of the sites. We refer to the two sampling designs as the multiple detection state model and multiple detection method model, respectively. In both cases a site must be occupied for certain detections to be recorded, but there is some possibility when an uncertain detection is recorded that the site is actually unoccupied (i.e., false positive detection).

We estimate occupancy dynamics among seasons following the general framework for multiseason occupancy models described by MacKenzie et al. [25], [26]. The model is comprised of three types of parameters: 1) the initial state distribution, 2) the between season transition probabilities, and 3) the detection probabilities. In the simplest case with two occupancy states (where a site is either occupied or not occupied), the initial probability of a site being occupied in time 0 is denoted by ψ_0_ and the probability of being unoccupied by 1−ψ_0_. The probability an unoccupied site in time *t* will be occupied in time *t* +1 is γ_t_ and the probability it will remain unoccupied is (1−γ_t_). Similarly, the probability an occupied site in time *t* will be unoccupied in *t* +1 is ε_t_ and the probability it will remain occupied is (1−ε_t_).

The difference between our approach and the approach described by MacKenzie et al. [25] for dynamic occupancy estimation is in how detection probabilities are formulated. First consider the case with multiple detection states where both uncertain and certain detections can occur during a single sampling occasion. For unoccupied sites, by definition certain detections do not to occur, thus, only two possible observations can occur: an uncertain detection or no detection. The probability of a false positive detection occurring for an unoccupied site is *p*_10_ and the probability of no detection is 1−*p*_10_. For occupied sites, no detections, certain detections, and uncertain detections can occur. We use 1−*p*_11_ to denote the probability of not detecting the species. The probability the detection will be certain, *b*, is conditional on detecting the species at an occupied site. The probability of an uncertain detection is *p*_11_*(1–*b*) and of a certain detection is *p*_11_*b.

Now consider the multiple detection method design where individual sampling occasions will include either all certain detections or all uncertain detections. When the uncertain method is used, species will be detected at sites that are unoccupied with a false positive detection probability *p*_10_, while no detection will occur with probability (1- *p*_10_). For occupied sites, the true positive detection probability is *p*_11_ and the probability of a false negative error is (1- *p*_11_). When the certain method is used, the probability is 1 that no detections will occur for unoccupied sites. If the site is occupied, the probability of a true positive detection is *r*_11_ and of not detecting the species is (1- *r*_11_).

The parameters above can be used to calculate the probability of an encounter history occurring for a site, where **h** ***_i_*** is the encounter history for the *i* ^th^ site. The product of the probabilities for data from all the sampled sites is then used to generate maximum likelihood estimates for parameters. The following provides examples of how to calculate probabilities for different potential encounter histories. Consider the case where a site is sampled on 3 occasions during each of two consecutive seasons. When both types of observations can be recorded in the same sampling occasion, we denote non-detections as 0, uncertain detections as 1, and certain detections as 2. The interval between seasons is shown using a space so that the encounter history **h = **000 101 means that the species was not detected at all during the first season, and uncertain detections were recorded in the first and third occasions during the second season. The likelihood of this encounter history occurring is:
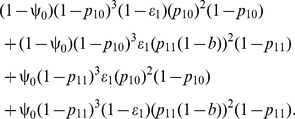



Because no certain detections occurred, it is possible that the site could have either been occupied or unoccupied in each of the two time periods. Thus, the probability is the sum of the probabilities for each of the 4 possible state combinations: unoccupied in both seasons, unoccupied then occupied, occupied then unoccupied, or occupied in both seasons. In each case the probability calculation is the product of 1) the probability of being in the initial state, 2) the probability of observing a set of detections conditional on the starting state, 3) the probability of being in a state in the second season conditional on the initial state, and 4) the probability of observing a set of detections conditional on the state of the site in the second season. Calculating probabilities of encounter histories that include additional seasons involves iterating the 3^rd^ and 4^th^ steps for each additional season.

Consider another site where the observed encounter history is **h = **210 011, that is both certain and uncertain detections occurred during the first season, but only uncertain detection occurred in the second. The probability of this encounter history is given by
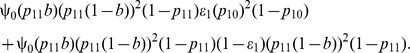



Because a certain detection occurred during the first season we have only two possibilities for the true state of the site over the two seasons. The first possibility is that the site was occupied in the first period but transitioned into being unoccupied in the second. In this case detections during the second season would be false positives. Alternatively, the site could have been occupied in both seasons.

Next consider a survey involving two detection methods employed on separate occasions, where the site is sampled twice using an uncertain method and once using a certain detection method in both seasons. The encounter history **h = **00/0 11/0 means that no detections were recorded in the first season with either method while two uncertain detections were recorded in the second. Because no certain detections were recorded, all 4 possible combinations of unoccupied and occupied states for the 2 years are again possible. The probability of the encounter history is given by:
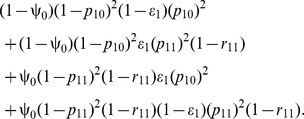



Note that the detection portion of the probabilities only has 2 terms for unoccupied sites. This is because the probability of getting a non-detection for the second survey conditional on the site being unoccupied is 1.

Next consider the encounter history **h = **10/1 11/0 where a certain detection occurs in the first season. The probability of the history is:
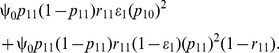



Because of the certain detection during the first season, only two possibilities exist for the true states during the two seasons, occupied in the first season and not in the second or occupied in both.

Further variation can be accounted for by allowing any of the parameters to vary among seasons, among sampling occasions, or among sites. This is easily done by specifying model parameters as linear functions of covariates (e.g. logit[ε*_it_*] = **β**′**X** *_it_*).

### General Approach

We can formulate the estimator for a general sampling design allowing for additional occupancy and observation states and for varying degrees of certainty. Sampling designs in which observations can be divided into certain and uncertain detections are a special case of the more general estimator described here. Multistate occupancy models that allow for >2 occupancy states are useful for many sampling situations [3], [26], [27]. Similarly extending the number of possible observation states (i.e., the set of discrete observations that can be made during a visit to a site) may be useful both to encompass additional occupancy states and differences among observation types in their probabilities of occurrence. In the second case we can relax the general assumption of a one to one match between occupancy states and observation states. The utility of such an approach is illustrated by our example where both certain and uncertain detections occur in the same sampling occasion. In this case 3 observation states correspond to two detection states. A more general approach will also be useful in conditions in which not all detections are either certain or uncertain, but instead detections vary in their degree of certainty [20]. The standard occupancy estimator [25], multistate estimator [26], simple mixture model with false positives [19], and multiple detection state and method models (here and [20]) are all special cases of the general formulation shown here. The overall approach we describe is analogous to the multievent modeling used for mark-recapture data [13].

We consider a standard multiseason occupancy survey where *n* sites are monitored for T seasons. The *i* ^th^ site is visited *R_it_* times during the *t* ^th^ season. The true occupancy state of the *i* ^th^ site in the *t* ^th^ season, *z_it_*, is one of *K* discrete occupancy states. Observations of the *i* ^th^ site on the *r* ^th^ visit in the *t* ^th^ season, y*_irt_*, are classified into one of *L* observation states that differ in the probability of being a false positive detection. The probability of recording an observation conditional on the true occupancy state is π*_lk_* = Pr(y*_irt_* = *l*|z*_it_* = *k*). In cases where a second sampling method is employed, the *n* sites are visited *S* additional times. Observations of the *i* ^th^ site on the *s* ^th^ visit in the *t* ^th^ season, w*_ist_*, are classified into one of *M* observation states. The probability of detecting a species for the second method, conditional on the true occupancy state is τ*_mk_* = Pr(w*_ist_* = *m*|z*_it_* = *k*).

The likelihood of the full set of parameters θ given the full set of encounter histories **H** can be formulated using the general likelihood for multiseason occupancy (MacKenzie et al. 2003, 2009). The likelihood is made up of 3 components: the initial state distribution, transition probabilities among states, and state specific detection probabilities. The full equation for the likelihood is
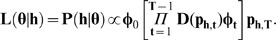



The initial state distribution 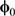 is a vector of length *K* where the *k* ^th^ element of the vector is the proportion of sites in the *k* ^th^ occupancy state at the start of the study. Transition probabilities among occupancy states between years are given by the *K* by *K* matrix 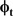. The element of the transition probability matrix in the *a* ^th^ column and *b* ^th^ row give the probability a site will be in occupancy state *b* in time *t* +1 given it was in state *a* in time *t*.

The final component is **p_h,t_**, which is a vector with one element for each state that gives the probability of observing an encounter history **h** for year ***t*** conditional on being in that state. **D(p_h,t_)** is a diagonal matrix with **p_h,t_** in the diagonal elements and 0′s in all other elements. These detection probabilities are where our application differs from previous multiseason occupancy models by allowing that false positives may occur.

The *k* ^th^ element of **p_h,t_** is the product of the probabilities of the observations occurring for each occasion of the encounter history given the true state of the site is *k*. In the case where a single detection method is used, the *k* ^th^ element is given by
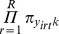
and when two methods are used the *k* ^th^ element is given by



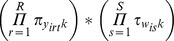



The same likelihoods can be used to implement the detection structure of other single season occupancy estimators that account for false positives to be used in dynamic analyses. In addition, the approach is flexible enough to allow for other multistate occupancy models that incorporate multiple species, abundance classes, reproductive state, and habitat to be modified to allow for false positive detections.

### Example: Range Dynamics of Gray Wolves

We use the estimator to examine multiseason occupancy patterns of gray wolves in northern Montana from 2007–2010. The area includes Montana’s portion of the federally designated Northwest Montana Recovery Area [28]. We rely on two sources of data: observations of wolves by hunters collected during telephone surveys used by the state to estimate deer and elk harvest (i.e., uncertain method; [29]–[31]) and known locations of resident wolf packs collected using radio-telemetry based monitoring of marked individuals (i.e., certain method; see [32]). Hunter survey data offer a wealth of information about the occurrence and distribution of wolves in the state, representing millions of hours of time spent in the field each year. However, these data were likely to include observation errors due to observer inexperience and the sensitivity of hunters to the recent political controversies concerning wolves. Alternatively, the known locations of radio-collared wolves represent an incomplete but irrefutable sample of occurrence locations from the wolf population. We show how our estimator can be used for the combined data to make inferences not possible using either data set alone.

We make a few key assumptions in using our approach. First, certain detections only occur for established packs and thus our estimates of occupancy are for the probability a pack occurs rather than wolves in general. We suspect that in some cases hunters report transient wolves, which would be the functional equivalent of false positive observations in our model. This definition of occupancy is concurrent with our desired metric for monitoring, making it a feature of the approach in our case. We also work under the assumption that the probability a pack is detected using the certain survey method (i.e., the probability that >1 wolf from a pack is captured and radio-collared) is not correlated with detection probabilities of packs by hunters. We believe our sample of known packs is representative and this was not an issue but consideration should be given to this condition when using our estimator. Finally, locations for our certain method of observation are typically are gathered before and immediately after the hunting season rather than during the hunting season. We therefore assume that the resident wolf packs monitored for our certain detection method use the same territories during the hunting season.

Our goal was to estimate the proportion of 600-km^2^ grid cells (i.e., mean territory size of wolf packs in Montana; [30]) that were used by wolf packs during the late fall and how occupancy in each year was influenced by the previous occupancy status of the sites. Sampling occasions for the hunter surveys were based on temporal replication. We treated each week of the 5-week general rifle season as a separate observation occasion. Hunters were asked to report where and when they saw wolves and this information was used to assign detection to grid cells during each of the sampling occasions [31]. We only had a single observation occasion each year for the known pack location survey. We considered a detection to have occurred if the centroid of radio-collar locations during a season was within a cell. This was a conservative measure of occurrence and allowed us to give equal weighting to packs with intensive location data (e.g., animals with GPS collars) and those where monitoring was more intermittent and based on one or a few locations during the sampling period.

**Figure 1:**
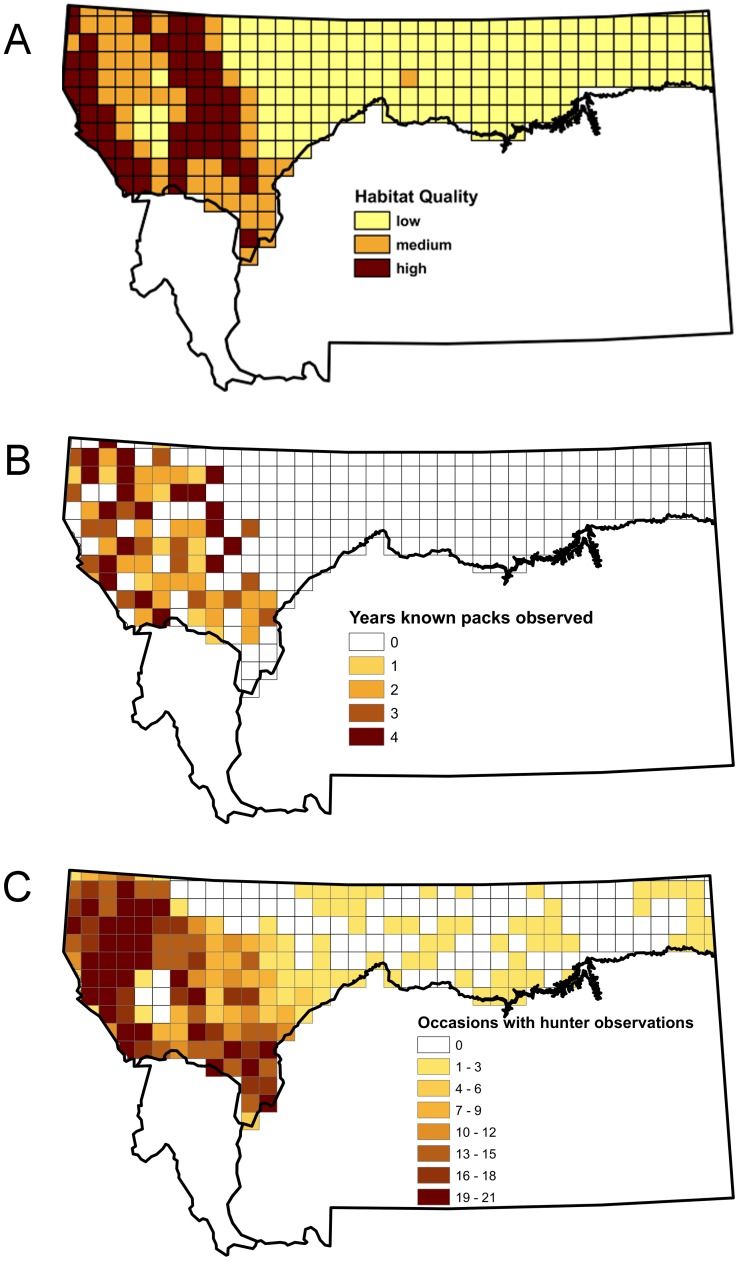
We estimated the proportion of grid cells in northern Montana that were occupied by wolves. Cells were divided based on perceived habitat quality into low, medium, and high quality categories (A). Most certain observations of known packs based on collaring and relocation by radio telemetry were concentrated in the western end of the study area where the higher quality habitat was located (B). While high frequency of observations by hunters also occurred in high-quality areas, they also reported a low frequency of wolf observations in the eastern portion of the study area, which we suspected were due to misidentification (C).

We recognized there was wide variation in the density of wolves related to habitat quality across this region and classified cells based on a composite measure of habitat quality. Based on prior research we identified 4 measures we believed to be good predictors of wolf distribution [30], [31]: the proportion of forest cover, mean elevation, mean slope, and a measure of terrain ruggedness of cells. All of these were strongly positively correlated making it impossible to separate the influence of each. Rather than arbitrarily selecting one measure, we created a composite predictor that equally weighted all of them. We z-transformed values of all 4 and summed them to generate a single habitat covariate H. We classified cells as low-quality if H was less than average (H <0; n = 168 cells), high-quality if the average of the habitat covariates was greater than 1 SD above the mean (H >4; n = 59), and medium quality if H was intermediate (0< H <4; n = 54; Figure 1).

Unaccounted-for heterogeneity in observation effort can bias estimates of occupancy making it important to account for variation in effort [1], [11]. Observer effort, which should be related to both true positive and false positive detection probabilities, will be a function of the amount of time spent by hunters in an area. Based on telephone surveys completed by hunters each year, we estimated the number of deer and elk hunter days per km^2^ in each of the 162 hunting units within Montana using a stratified network estimator [29]. We assigned a value for the summed estimate of deer and elk hunter days to each cell according to the hunting unit in which it occurred. Areas such as national parks and tribal reservations where hunting did not occur were treated as missing values to correctly account for the fact that sampling did not occur in these areas.

All models were fit using PRESENCE v 3.1 [33]. We used Akaike’s Information Criterion (AIC) to choose among alternative parameterizations of the model allowing for false positive detections. First, we selected among alternative parameterizations for detection parameters (p_11_, p_01_, and r_11_), where the most general parameterization for other parameters was used (model 5 in the next paragraph). We considered four alternatives that differed according to whether detection for the hunter survey (p_11_ and p_01_) varied with respect to hunting effort and whether the known-pack survey locations (r_11_) varied by habitat quality. The detection models were: 1) neither effect, 2) only the effort effect, 3) only the habitat effect, and 4) both effects. In all cases we specified that all detection parameters varied among years. Hunter effort was used to account for the wide variation among grid cells in the opportunity for hunters to detect wolves, which was likely to affect detection probabilities. We included an effect of habitat quality to account for the possibility that a perceived lack of wolves in low-quality cells may have led to lower detection rates of established packs.

Using the parameterization for the best detection model (lowest AIC), we considered 5 alternatives for the occupancy parameters related to whether or not transitions (ε and γ ) varied annually and whether all the parameters (ψ_0_, ε, and γ) varied by habitat quality. The alternative occupancy models included: 1) neither effect, 2) only the year effect, 3) only the habitat effect, 4) an additive combination of habitat and year, and 5) an interaction between habitat and year.

We also compared the estimates from the model with the lowest AIC among our alternatives to equivalent parameterizations where false positives were assumed not to occur and where both false negatives and false positives were assumed not to occur. First, we estimated parameters when false negatives were allowed but false positives were not. We did this by fixing the false positive probability to equal 0 in the models described above. This is equivalent to using the standard dynamic occupancy estimator proposed by MacKenzie et al. [25]. In addition, we generated naïve estimates assuming that false negatives and false positives did not occur. To do this we assumed that wolves were present if they were detected at least once during either survey type and estimated initial occupancy probabilities and transition probabilities based on this assumption. We were able to generate naïve estimates from this data set using Presence. We fixed the false positive probability to 0 and the true positive detection probability to 1 and generated encounter histories with a single visit per season where cells with no detection were assigned a zero and cells with at least 1 detection by any method was assigned a 1.

## Results

**Figure 2:**
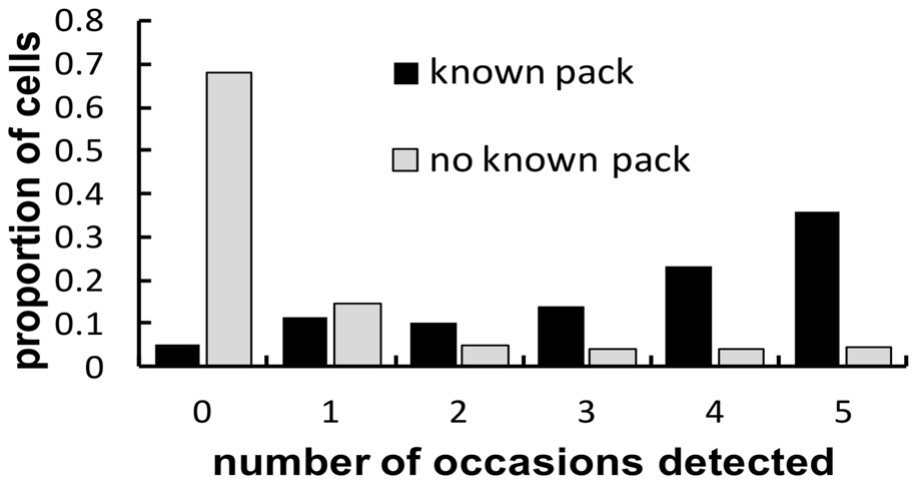
The proportion of cells with and without known packs where wolves were observed by hunters from 0 to 5 times out of the 5 sampling occasions that occurred in each year. For cells with known packs this distribution increased across the possible values peaking at the maximum of 5 times. For cells without a known pack, as expected due to unoccupied cells, the proportion of 0 observations was much higher than for those without a known pack. If no false positives occurred we would expect the relative frequencies for 1 to 5 hunter observations to be the same for cells with and without known packs. Instead we see a greater relative frequency of 1 or 2 observations in cells without known packs, which is consistent with a low probability of false positive errors occurring in unoccupied cells.

In general, medium and high habitat quality cells were in the western end of the study area and are associated with mountainous and forested terrain (Fig. 1A). Cells where a known pack was observed in at least one year are concentrated in the western end of the study area in the higher habitat quality areas (Fig. 1B). Observations of wolves by hunters occurred in other parts of the study area, although in most cases wolves were observed in these areas during only a few occasions (Fig. 1C). Differences in the distribution of observations for cells with and without known packs were consistent with our expectations if false positives occurred (Fig. 2). The occupancy models we fit allowed us to determine the support for two alternative explanations for these patterns in the data. The first was that misidentification was leading to false positive errors in the eastern part of the state, while the other was that low numbers of hunter detections and few known packs were a result of lower hunter effort and fewer collared animals in what was perceived to be low quality habitat. Determining which of these had greater support has important implications for understanding the current range extent of wolves in the state.

The parameterization of our model with the lowest AIC value was the one where detection for the hunter survey increased with respect to hunter effort and detection of packs was somewhat lower in low quality habitat (detection model 4). The best model for initial occupancy and transitions included an additive function of year and habitat quality (occupancy model 4). The next best model, which did not include an annual effect on transition probabilities, had ΔAIC = 6.3. Because of the strong support for the best model and for ease of comparison we focused on results for the best fitting model.

**Figure 3:**
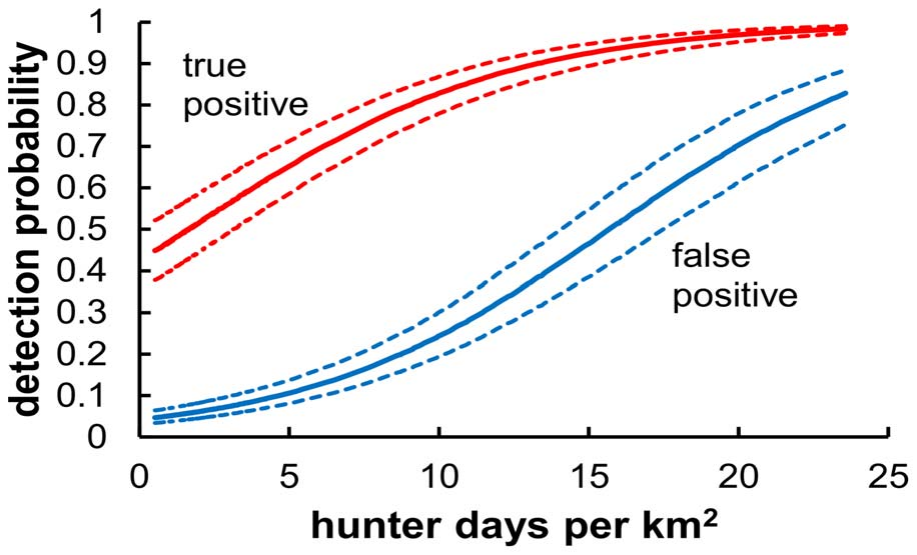
Both true positive and false positive detection probabilities increased with increased hunter effort as measured at the hunting unit level by the number of hunter days in the field per km^2^. Plotted lines are the estimated relationships for 2010, and dashed lines are 95% confidence intervals. Our best model included an additive effect of year so that in other years detection had the same basic relationship to hunter effort on a logit scale but was lower overall.

Both the true positive (p_11_) and false positive (p_01_) detection probabilities for the hunter survey data increased as hunter effort increased (Figure 3). False positive probabilities were greater than 0.5 in areas with the greatest hunting effort. A value of 0.5 would correspond with unoccupied cells having a 97% chance of at least 1 false positive detection during the 5 sampling occasions in a year. True positive detection probabilities for the certain survey (r_11_) were lower in low quality habitats than medium and high quality, and all detection probabilities increased across years.

**Figure 4:**
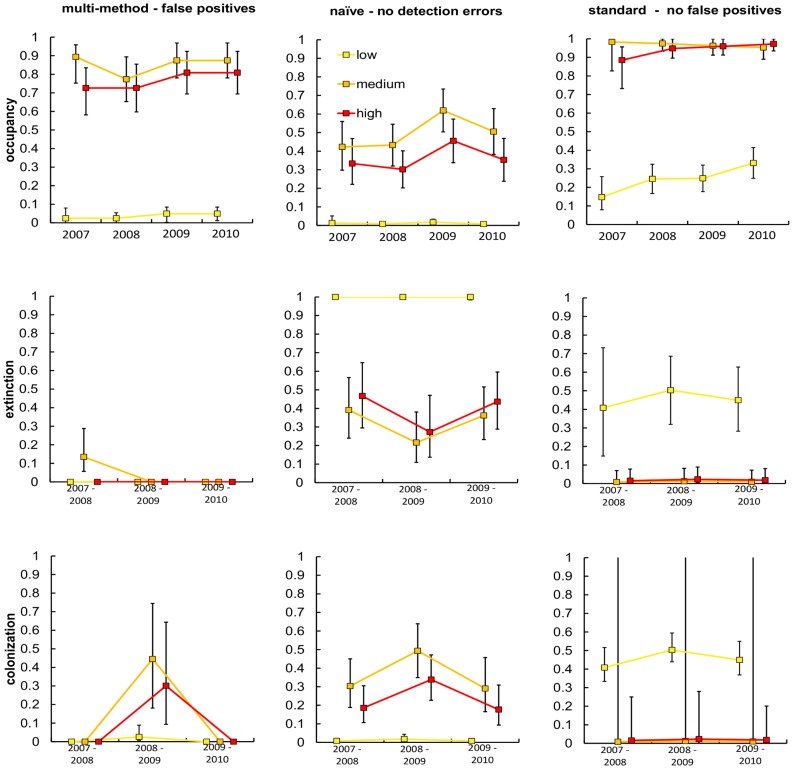
Estimates of occupancy, extinction, and colonization for gray wolves in northern Montana from 2007–2010. Cells were divided into low, medium, and high habitat quality. Parameters were estimated using a naïve approach where false positives and false negatives were assumed not to occur, using a standard multi-season occupancy estimator where false positives were assumed not to occur, and using our multi-season occupancy estimator that allows for false positives.

As expected, occupancy dynamics varied among low, medium, and high quality cells (Figure 4). Medium quality cells had slightly higher occupancy than high quality, although differences were small compared to the consistently lower occupancy estimated for cells with low habitat quality. The medium and high categories could probably be combined for future work. Occupancy was similar across all years due to the low probability of transitions across all habitats. Our best model included annual differences in transition probabilities. Our best estimate was that no extinction occurred from 2008–2009 and 2009–2010, and the only extinctions occurred in medium quality cells from 2007–2008. Colonization also appeared to increase for 2008–2009 from a negligible level for 2007–08, only to fall again in 2009–10.

Estimates based on our approach differed significantly from estimates using a naïve estimator and occupancy estimates when false positives were assumed not to occur (Fig. 4). Our model had a much lower AIC value than the traditional approach where false positives are assumed not to occur (ΔAIC = 443.2). Differences in the estimator structure did not allow for a similar comparison to the naïve estimates. Occupancy estimates were lowest and extinction and colonization probabilities highest for the naïve estimator. Occupancy estimates were greatest for the standard estimator that assumed that false positives did not occur. Occupancy in the medium and high quality cells was estimated to be near 100%, and estimates of occupancy in the low quality cells, which make up the majority of the study area, were moderate and rapidly increasing across the study period based on the standard estimator. Estimates of extinction and colonization probabilities were near 0 in the medium and high quality cells for the standard estimator, but high transition probabilities were still estimated for the low quality cells. Because almost all sites in the medium and high quality habitats were occupied there was little opportunity to estimate colonization rates. This is reflected in the large standard errors for these parameters. Occupancy estimates using our approach were in between those for the other methods with high occupancy in the medium and high quality cells and very low (≤0.04) and stable occupancy in the low quality cells. Overall transition probabilities were lowest when we accounted for false positives.

## Discussion

We demonstrate that detection errors in general, and false positives in particular, can have large effects on estimates of range-dynamics and other presence-absence processes. False negatives led to underestimation of occupancy, while false positives led to over-estimation of occupancy, extinction, and colonization. Whereas the importance of accounting for false negative errors is frequently recognized, much less attention has been given to the potential effects of false positive errors. However, even small probabilities of false positive errors can lead to significant over-estimation of occupancy once false negatives are accounted for [20]. This is illustrated by comparing our results for the low-quality cells to those of standard occupancy estimators. Estimates which do not account for false positives were 3–6 times greater in each of the years than those that did account for false positives. Further, standard occupancy estimates inferred that wolf occupancy was increasing in low-quality habitats, whereas estimates from the methods described here did not. Independent work on wolf population dynamics [32], as well as comparison of the current known wolf distribution with other wolf habitat models [33], indicates that wolf population growth in Montana may be slowing as suitable habitat is becoming filled. Accounting for false positives is that much more important for rare species with low occurrence probabilities [20], as is the case with wolves in low-quality habitat.

The effects of false positives are less predictable when estimating transition probabilities among time periods [11], [21]. This unpredictability makes it even more important to account for false positives (and negatives) when occurrence dynamics are of interest. In our study, unaccounted-for detection errors led to higher estimates of both colonization and extinction. In other cases unaccounted-for false positives will lead to underestimation of transition probabilities (DAWM unpublished data).

Our results in general suggest that hunter observations are a viable survey method to monitor range dynamics of wolves, particularly when detection errors are accounted for. We found that range size was generally stable across the years of our study, with most occurrences restricted to higher quality habitat in the western end of our study area, consistent with existing models of wolf habitat suitability [34]. Interestingly, we found that during the 3 annual transition periods measured in this study, turnover of occurrence status at the site level was rare. Occupied areas almost always remained occupied, and unoccupied areas were colonized with low probabilities. This indicates a high degree of stability at the distribution level, suggesting perhaps that wolves now occupy most of the highest quality habitat in northern Montana.

In recent years initiatives to collect and analyze large and extensive data sets collected by the public have increased [35]. Too frequently, analyses of these data give little consideration to observation uncertainty and in the worst cases are conducted without properly considering the sampling effort that generates the data [36]. In many cases it is possible to address issue of sampling effort and detection in analyses using existing data. Excellent examples exist for efforts to address non-detection in large-scale public surveys [5], [6], [10]. Previous efforts to deal with false positive errors, however, have largely relied on ad-hoc approaches to reduce their occurrences in data sets (e.g., [7], [22]). Proper effort should be given to reducing false positives as part of study design. However, this is unlikely to eliminate errors completely [17], making it important to estimate and account for false positives as part of the statistical analysis. Methods such as ours will continue to improve the inferences for many large-scale endeavors where false positives are likely to be an issue.

False positive errors are not limited to data collected by the public, and growing evidence suggests they may be common to many ecological data sets. Multiple studies have demonstrated that false positives frequently occur in auditory call surveys for birds and amphibians [11], [17], [37]–[41]. False positives are not limited to human observers but also occur in data from computer sound analysis of audio files from automatic recording devices [18], [42]. Indirect observations of species such as scat and tracks may also be prone to false positive errors [7]. In the case of cryptic or variable species, false positives can be common even when organisms are identified under close visual examination [16]. False positives are recognized to occur for many laboratory assays and often estimated (i.e., sensitivity; [21]). The methods we propose here are by no means limited in applicability to public surveys and should be useful for studies that use many of these other data types.

The emergence of large collaborative monitoring efforts is an exciting development that will provide many unique opportunities to inform conservation and improve ecological understanding. The success of these efforts will depend on whether analysis methods properly account for the observational uncertainty that is inherent in these data sets. The methods we present here are an important step in that direction. We believe explicitly accounting for observational uncertainty can address the limitations of many data and will open the door to many exciting applications.
